# Dietary vitamin D intake and changes in body composition over three years in older adults with metabolic syndrome

**DOI:** 10.1016/j.jnha.2024.100467

**Published:** 2025-01-08

**Authors:** Héctor Vázquez-Lorente, Jiaqi Ni, Nancy Babio, Ana García-Arellano, Dora Romaguera, J. Alfredo Martínez, Ramon Estruch, Vicente Martín Sánchez, Josep Vidal, Montserrat Fitó, Maira Bes-Rastrollo, Jadwiga Konieczna, Diego Martinez-Urbistondo, Rosa Casas, Marcos García-Fernández, Romina Paula Olbeyra, Alice Chaplin, M. Angeles Zulet, Jordi Salas-Salvadó

**Affiliations:** a Universitat Rovira i Virgili, Departament de Bioquímica i Biotecnologia, Alimentació, Nutrició, Desenvolupament i Salut Mental ANUT-DSM, Reus, Spain; b Institut d'Investigació Sanitària Pere Virgili (IISPV), Reus, Spain; c Centro de Investigación Biomédica en Red Fisiopatología de la Obesidad y la Nutrición (CIBEROBN), Institute of Health Carlos III, Madrid, Spain; d Department of Preventive Medicine and Public Health, Instituto de Investigación Sanitaria de Navarra (IdiSNA), University of Navarra, Pamplona, Spain; e Health Research Institute of the Balearic Islands (IdISBa), Palma de Mallorca, Spain; f Department of Nutrition, Food Sciences, and Physiology, Center for Nutrition Research, University of Navarra, Pamplona, Spain; g Precision Nutrition and Cardiometabolic Health Program, IEA Food, CEI UAM + CSIC, Madrid, Spain; h Departamento de Medicina y Endocrinología, Universidad de Valladolid, Spain; i Department of Internal Medicine, Institut d'Investigacions Biomèdiques August Pi Sunyer (IDIBAPS), Hospital Clinic, University of Barcelona, Institut de Recerca en Nutrició i Seguretat Alimentaria (INSA-UB), Barcelona, Spain; j Centro de Investigación Biomédica en Red | Epidemiología y Salud Pública (CIBERESP), Instituto de Salud Carlos III, Madrid, Spain; k Institute of Biomedicine (IBIOMED), University of León, León, Spain; l CIBER Diabetes y Enfermedades Metabólicas (CIBERDEM), Instituto de Salud Carlos III (ISCIII), Madrid, Spain; m Department of Endocrinology, Institut d`Investigacions Biomédiques August Pi Sunyer (IDIBAPS), Hospital Clinic, University of Barcelona, Barcelona, Spain; n Unit of Cardiovascular Risk and Nutrition, Hospital del Mar Research Institute (IMIM), Barcelona, Spain; o Centro de Salud Vidriales, SACYL, Santibañez de Vidriales, Zamora, Spain

**Keywords:** Vitamin D, Body composition, Fat mass, Lean mass, Aging, Older people

## Abstract

**Background:**

Adequate intake of vitamin D through diet may offer benefits in terms of body composition.

**Objectives:**

We aimed to evaluate the longitudinal relationship between dietary vitamin D intake and changes in body composition in older adults over one and three years under the context of a weight loss and lifestyle behavioral intervention.

**Design:**

Longitudinal study.

**Setting:**

Multicenter.

**Participants:**

This longitudinal study included 715 aged participants (mean age 65.3 ± 5.0 years, 38% women) with overweight/obesity and metabolic syndrome.

**Measurements:**

Multivariable-adjusted mixed-effects linear regression models were fitted to investigate the longitudinal associations between dietary vitamin D intake (exposure) and body composition (outcome) with available data at baseline, one, and three years of follow-up. Data on dietary vitamin D intake was assessed using a validated 143-item food frequency questionnaire. Body composition variables (total body weight (kg), total fat mass (%), total lean mass (%), muscle-to-fat mass ratio, visceral adipose tissue (kg), and android-to-gynoid fat ratio) were measured by dual-energy X-ray absorptiometry.

**Results:**

Higher dietary vitamin D intake (for each μg/day) was associated with higher total lean mass (β: 0.10 %; 95% CI: 0.02 to 0.18; P: 0.017) and muscle-to-fat mass ratio (β: 1.00 × 10^−2^; 95% CI: 0.22 × 10^−2^ to 1.78 × 10^−2^; P: 0.011), and lower total body weight (β: −0.20 kg; 95% CI: −0.34 to −0.05; P: 0.007), total fat mass (β: −0.11 %; 95% CI: −0.19 to −0.02; P: 0.015), and visceral adipose tissue (β: −1.74 × 10^−2^ kg; 95% CI: −3.47 × 10^−2^ to −0.01 × 10^−2^; P: 0.048) at one year of follow-up in the group following the intervention in the multivariable-adjusted model.

**Conclusion:**

Dietary vitamin D intake was associated with better body composition changes in the context of a weight loss and lifestyle intervention which led to notable changes in body composition at short term.

## 1 Introduction

Obesity is a complex and multifactorial condition primarily resulting from an interplay of genetic, psychological, socioeconomic, metabolic, environmental, and behavioral determinants [1]. Increasing rates of lifestyle-related obesity represent a major worldwide challenge in public health [2]. Consequently, the identification and implementation of prevention strategies targeting modifiable risk factors are of critical importance for the effective prevention and control of obesity [3].

Adequate lifestyle behaviors are well-established factors with the potential to prevent or modulate obesity [2]. Weight loss and lifestyle interventions, based particularly on diet and physical activity modifications, are suggested to be the cornerstone for the prevention and treatment of sarcopenic obesity in older adults [4], an undesirable and harmful condition affecting one in ten older adults in developed countries [5]. Concretely, dietary habits play a crucial role in promoting overall health and preventing weight gain, as certain dietary components may modulate body composition in obese individuals [6]. Among multiple nutritional factors, vitamin D has gained attention for its potential role in body composition regulation, with higher vitamin D consumption being suggested to normalize obesity related biomarkers [7].

The consumption of vitamin D through supplements may offer benefits in improving body composition profile [[8], [9], [10]], particularly by increasing total lean mass and reducing total fat mass [11]. However, the current body of evidence examining the relationship between dietary vitamin D intake and body composition profile over time in humans remains limited, with a notable lack of well-designed longitudinal studies addressing this research question [12]. Despite cross-sectional studies reporting mixed results, with some showing statistically non-significant relationship [13,14], and others indicating associations with better body composition [15,16], to our knowledge, no studies have longitudinally evaluated the relationship between dietary vitamin D intake and changes in body composition.

We previously demonstrated improvements in body weight and composition after one year that were attenuated after three years of follow-up under a weight loss and lifestyle intervention based on an energy-reduced Mediterranean diet with physical activity promotion compared to an *ad libitum* Mediterranean diet in the same subsample [17]. However, whether dietary vitamin D intake, a micronutrient which is an important component of the Mediterranean diet [18], is associated with these changes in body composition remains unknown. The present study aims to assess longitudinal associations between dietary vitamin D intake and changes in body composition under the context of a weight loss and lifestyle intervention in a cohort of aged individuals with overweight/obesity and metabolic syndrome over one and three years of follow-up.

## 2 Materials and methods

### 2.1 Study design

This longitudinal study was performed within the framework of the PREDIMED-Plus study, an ongoing multicenter Spanish randomized controlled trial across 23 centers that aims to evaluate the effect of an energy-reduced Mediterranean diet, increased physical activity and motivation support on primary cardiovascular disease prevention [19]. Briefly, PREDIMED-Plus is an ongoing multicenter, parallel-group, randomized, single-blind clinical trial evaluating the long-term effects of a lifestyle intervention including an energy-reduced Mediterranean diet, physical activity promotion, and behavioral support for weight loss (intervention group) vs. a control group receiving general Mediterranean diet with *ad libitum* recommendations and without any caloric restriction on cardiovascular events and mortality. Further details regarding the trial protocol can be accessed at https://www.predimedplus.com/ and in previously published sources [20,21]. Ethical approval was obtained from all participating centers, and written informed consent was obtained from all participants. The trial was registered in 2014 at the International Standard Randomized Controlled Trial registry [ISRCT; www.isrctn.com/ISRCTN89898870].

### 2.2 Participants

**Fig. 1:**
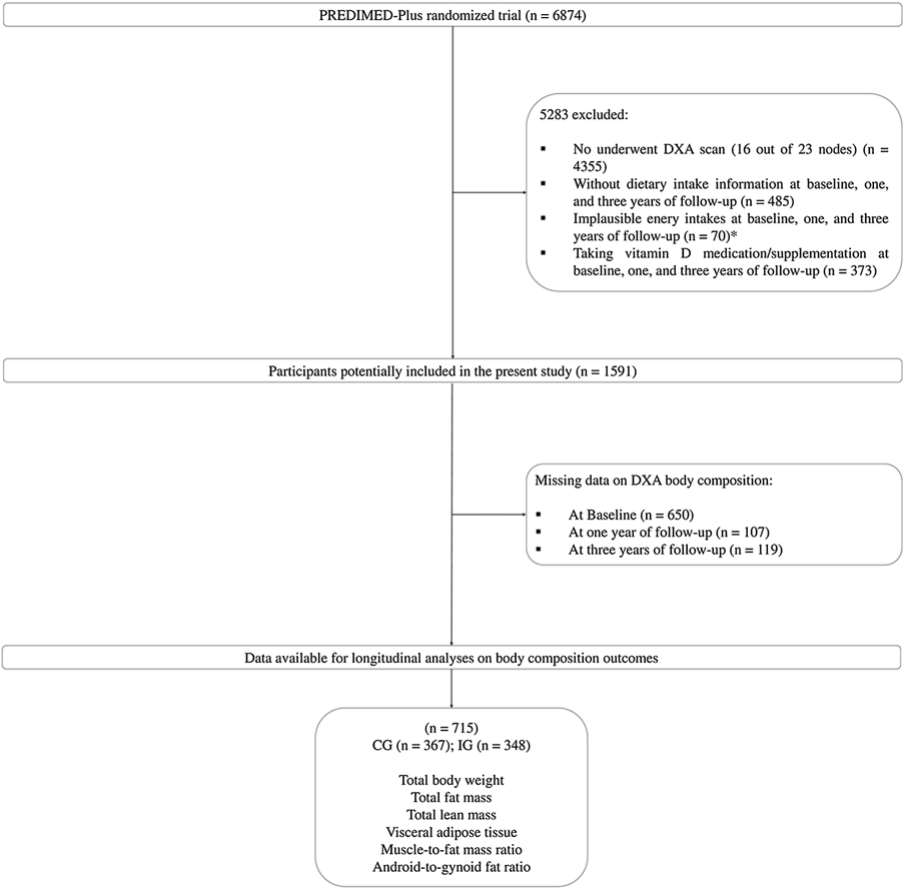
Flowchart of the study population. Abbreviations: CG, control group; IG, intervention group. *Daily energy intakes for men <800 kcal or >4000 kcal and women <500 kcal or >3500 kcal [23].

The study enrolled community-dwelling adults aged 55–75 years with overweight or obesity (body mass index (BMI) ranging from 27 to 40 kg/m²) who met at least three criteria for metabolic syndrome, as defined by established criteria [22]. Exclusion criteria were based on I) unwillingness to give written informed consent, II) institutionalization, III) pre-existing cardiovascular diseases, psychiatric disorders, or bowel diseases, IV) weight loss medication use, and V) inability to follow the intervention based on religious purposes, food allergies or intolerances, or injuries. From October 2013 to December 2016, a total of 6874 eligible participants were randomly assigned in a 1:1 ratio to either the intervention group or the control group receiving usual care (traditional energy-unrestricted Mediterranean diet). Randomization was centrally performed using a computer-generated random number internet-based system with stratification by center, sex, and age (<65 years, 65–70 years, and >70 years) in blocks of 6 participants. The randomization procedure was blinded to all staff members and principal investigators. For participant couples sharing the same household, randomization was done by cluster, with the couple as the unit of randomization. For the purposes of the present study, data from a subsample of participants who underwent dual energy x-ray absorptiometry (DXA) measurements in 7 of the 23 recruiting centers having access to DXA scanners (DXA sub study) was used. We additionally excluded those participants who not completed the food frequency questionnaire (FFQ), reported energy intakes falling outside predefined limits (<800 to ≥4000 kcal/d for men, <500 to ≥3500 kcal/d for women) [23], or consumed vitamin D medication/supplementation at baseline, one, and three years of follow-up. A total of 1591 participants were eligible for the present study, with 715 participants (mean age 65.3 ± 5.0 years, 38% women) presenting available information in all time points (Fig. 1).

### 2.3 Exposure: dietary vitamin D intake

Trained dietitians conducted face-to-face interviews with participants to assess their dietary habits using a validated 143-item FFQ [24]. The FFQ comprised of nine response options ranging from "never" to "more than six times per day." These responses were converted into daily intake values using standard portion sizes for each item. The Spanish food composition database was utilized to estimate the energy (kcal/day) and dietary vitamin D intake (μg/day) [25], which was assessed at baseline, one year, and three years of follow-up. The energy-adjusted dietary vitamin D intake was subsequently calculated using the residual method [26]. Intraclass correlation coefﬁcients for relative reproducibility and validity for energy-adjusted dietary vitamin D intake were 0.68 and 0.39, respectively.

Vitamin D medication/supplementation was self-reported by the participants when, at any point during the three-year study, they indicated consuming any type of compound containing vitamin D that was not coming from a dietary source. To dismiss the putative effect of medication and/or supplementation of vitamin D on body composition, all analyses were conducted excluding subjects taking medication and/or prescribed supplementation of vitamin D in all time points.

### 2.4 Outcome: body composition

Body composition variables were measured with a third-generation DXA scanner (DXA Lunar Prodigy Primo and Lunar iDXA; GE Healthcare) connected with enCore software by trained operators at baseline, one, and three years of follow-up. The body composition end points were total body weight in kilograms, total fat and lean mass expressed as the percentage of DXA-derived total body weight, and visceral adipose tissue expressed in kilograms. For visceral fat measurements in an android region, DXA scans were reanalyzed using CoreScan software application (GE Healthcare) validated using computed tomographic scans as a reference tool [27]. The regions of interest (ROI) for regional body composition measurement were defined using the software provided by the manufacturer. Among them, the abdominal android and gynoid ROI were obtained as previously described [28]. To deepen our understanding of body composition, the muscle-to-fat mass ratio as a marker of sarcopenic obesity and the android-to-gynoid fat ratio to account for age-related adiposity redistribution from central to peripheral regions were subsequently calculated.

### 2.5 Covariate assessments

Sociodemographic and lifestyle information regarding age, sex, education level, civil, and smoking status was collected through administered questionnaires. Height was measured without shoes in duplicate using a wall-mounted stadiometer. Physical activity was estimated utilizing a the validated REGICOR (Registre Gironí del Cor) Short Physical Activity Questionnaire for adult population adapted from the Minnesota Leisure Time Physical Activity Questionnaire (MLTPAQ) [29], whereas sedentary behavior was obtained using the validated Spanish version of the Nurses’ Health Study questionnaire [30]. Personal medical history, encompassing conditions such as type 2 diabetes, hypertension, and hypercholesterolemia, as well as medication usage, were either self-reported or extracted from medical records. Food groups information including variables related to the consumption of vegetables, fruits, legumes, cereals, meat, fish, dairy, nuts, oils and fats, olive oils, biscuits, coffee, tea, and alcohol was collected by the same FFQ used for assessing dietary vitamin D intake. Subsequently, energy and nutrient intake estimations were derived utilizing Spanish Food Composition Tables [25].

### 2.6 Statistical analyses

Main analyses were performed in the evaluable population including all available data in exposure and outcomes of participants at baseline, one, and three years of follow-up. Since an interaction by the intervention group was observed, all analyses were stratified according to the intervention group.

Baseline characteristics of the study cohort were presented both in overall participants and categorized by intervention group as means ± SDs for continuous variables, and numbers (percentages) for categorical variables. Unpaired Student’s t-test and chi-square test were employed for continuous and categorical variables, respectively. Unpaired Student’s t-test was additionally employed to assess differences in mean changes in body composition and energy-adjusted dietary vitamin D intake by study arm.

Linear mixed-effects models were used to assess relationships between energy-adjusted dietary vitamin D intake as a continuous variable (exposure) and changes in body weight and composition (outcome) at baseline, one year, and three years of follow-up by intervention group. Three-level linear mixed models were fitted with random intercepts at the recruiting center, cluster family (as couples from the same household were randomized together) and individual participants. An interaction term of energy-adjusted dietary vitamin D intake with time as continuous was included as fixed effects, as well as age (years) and sex (men/women) in the basic models. Baseline education level (primary or less, secondary, or college), civil status (single, divorced or separated, married, widower), height (cm), smoking status (current, former, or never), diabetes prevalence (yes/no), hypertension prevalence (yes/no), hypercholesterolemia prevalence (yes/no), and physical activity (METs min/day), sedentary time (h/day), alcohol consumption in g/day (and adding the quadratic term), food groups (consumption of vegetables, fruits, legumes, cereals, oils and fats, olive oils, biscuits, meat, fish, dairy, nuts [g/day], coffee and tea [mL/day]), at baseline, one, and three years of follow-up were additionally included as fixed effects in the multivariable-adjusted models. These models were presented as β-coefficients along with their corresponding 95% confidence intervals (CIs).

Post hoc stratified analyses were conducted for body composition outcomes by baseline categories of age (<65 and ≥65 years), sex (men and women), smoking habits (current or former smoker, or never smoker), and physical activity (<median and ≥ median) using the likelihood ratio test. An interaction term between time, energy-adjusted dietary vitamin D intake, and each potential effect modifier was included within multivariable-adjusted models.

All statistical analyses were conducted with Stata/SE version 14.2 (StataCorp LLC, College Station, TX, USA) using the PREDIMED-Plus study dataset updated to December 19, 2023. All graphs were plotted using GraphPad Prism software v.9.0 (GraphPad Software, San Diego, CA, USA). Statistical significance was defined as a two-tailed P-value <0.05.

## 3 Results

Since an interaction by the intervention group was observed, all analyses were stratified according to the intervention groups. Table 1 presents the baseline characteristics of the overall study population and by intervention groups. The energy-adjusted mean dietary vitamin D intake at baseline was 6.0 ± 3.2 μg/day in the control group and 5.9 ± 3.3 μg/day in the intervention group (P = 0.966) (overall mean: 6.0 ± 3.2 μg/day). No baseline differences by intervention groups were observed for the variables of the study (all P ≥ 0.05; Table 1). In terms of dietary intake, participants in the intervention group consumed lower quantities of total dairy products compared to those in the control group (P = 0.037; Table S1). No more intergroup differences were observed for the food groups and main macronutrients analyzed.

**Table 1:** Baseline characteristics of the PREDIMED-Plus participants (overall and by intervention groups).

	Total population (n = 715)	Control group (n = 367)	Intervention group (n = 348)	P-value^1^
Dietary vitamin D intake (μg/day)	5.9 ± 3.2	6.0 ± 3.2	5.9 ± 3.3	0.966
Height (m)	164.0 ± 9.1	163.8 ± 9.2	164.3 ± 9.0	0.454
Sociodemographic variables				
Age (years)	65.3 ± 5.0	65.4 ± 4.9	65.2 ± 5.2	0.564
Women (n/%)	272 (38.0)	136 (37.1)	136 (39.1)	0.578
Education level (n/%)				
Primary or less	331 (46.3)	179 (48.8)	152 (43.7)	0.247
Secondary	226 (31.6)	106 (28.9)	120 (34.5)	
College	158 (22.1)	82 (22.3)	76 (21.8)	
Civil status (n/%)				
Single, divorced or separated	80 (11.2)	42 (11.4)	38 (10.9)	0.159
Married	570 (79.7)	299 (81.5)	271 (77.9)	
Widower	65 (9.1)	26 (7.1)	39 (11.2)	
Disease presence or medication usage at recruitment				
Type 2 diabetes (n/%)	209 (29.2)	105 (28.6)	104 (29.9)	0.708
Hypertension (n/%)	606 (84.8)	319 (86.9)	287 (82.5)	0.098
Hypercholesterolemia (n/%)	473 (66.2)	251 (68.4)	222 (63.8)	0.194
Medication use (n/%)				
Insulin or other antidiabetic drugs	164 (22.9)	82 (22.3)	82 (23.6)	0.698
Antihypertensive agents	573 (80.2)	304 (82.8)	269 (77.3)	0.064
Statins or other hypolipidemic drugs	373 (52.2)	197 (53.7)	176 (50.6)	0.406
Lifestyle variables				
Physical exercise (METs min/day)	417.7 ± 13.2	439.9 ± 358.4	394.2 ± 344.3	0.083
Sedentary time (h/day)	5.9 ± 1.8	5.9 ± 1.9	5.8 ± 1.8	0.543
Smoking status, n (%)				
Current smoker	103 (14.4)	46 (12.5)	57 (16.4)	0.240
Former smoker	339 (47.4)	183 (49.9)	156 (44.8)	
Never smoker	273 (38.2)	138 (37.6)	135 (38.8)	
Body composition				
Total body weight (Kg)	85.8 ± 12.3	85.6 ± 12.6	86.1 ± 11.9	0.689
Total fat mass (%)	40.5 ± 6.7	40.3 ± 6.5	40.7 ± 6.9	0.620
Total lean mass (%)	59.5 ± 6.7	59.6 ± 6.5	59.3 ± 6.9	0.470
Muscle-to-fat mass ratio	1.5 ± 0.4	1.5 ± 0.4	1.5 ± 0.4	0.611
Visceral adipose tissue (Kg)	2.4 ± 0.0	2.4 ± 0.9	2.32 ± 0.88	0.140
Android-to-gynoid fat ratio	0.8 ± 0.2	0.8 ± 0.2	0.8 ± 0.2	0.108

Abbreviations: METs, metabolic equivalents.

Data are presented as n (%) or mean ± SD for categorical and continuous variables, respectively.

1 P value for differences intergroups was calculated by Pearson’s Chi-square test or unpaired student t-test, as appropriate.

The evolution of body composition variables of the study and dietary vitamin D intake separately in each study arm are represented in Table S2, Figure S1, and Figure S2. Compared to the control group, a decrease in total body weight, total fat mass, and an increase in total lean mass and muscle-to-fat mass ratio was shown at year one (all P ≤ 0.001) and three (all P ≤ 0.025) of follow-up in the intervention group, changes which were more remarkable at year one compared to those occurred at three years of follow-up. Lower visceral adipose tissue, and android-to-gynoid fat ratio was also observed in the intervention group compared to the control group at year one (all P ≤ 0.028). Moreover, compared to the control group, higher dietary vitamin D intake was observed in the intervention group at year one (P = 0.001) and three (P ≤ 0.031), which were more remarkable at year one.

**Fig. 2:**
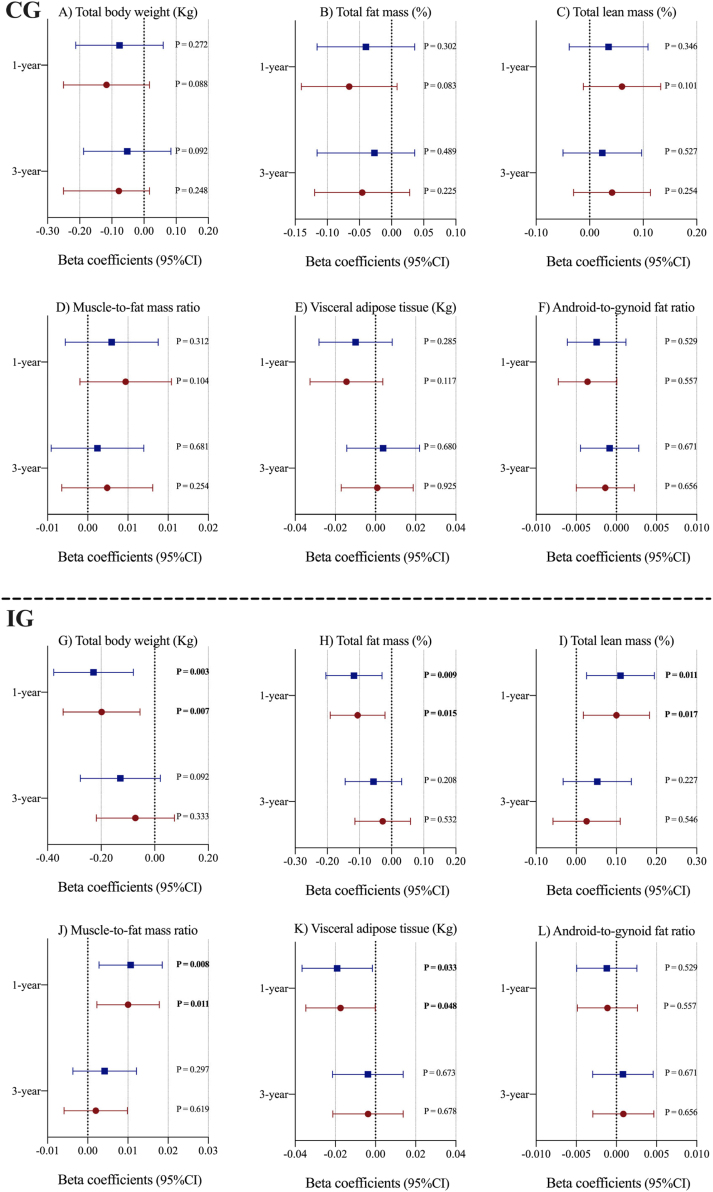
Longitudinal associations between energy-adjusted dietary vitamin D intake and changes in body composition over one and three years of follow-up in the PREDIMED-Plus cohort by intervention groups. Abbreviations: CG, control group; CI, confidence interval; IG, intervention group. Three-level linear mixed models were fitted with random intercepts at the recruiting center, cluster family (as couples from the same household were randomized together) and individual participants. An interaction term of energy-adjusted dietary vitamin D intake with time as continuous as well as age (years) and sex (men/women) were included as fixed effects in the basic models. Baseline education level (primary or less, secondary, or college), civil status (single, divorced or separated, married, widower), height (cm), smoking status (current, former, or never), diabetes prevalence (yes/no), hypertension prevalence (yes/no), hypercholesterolemia prevalence (yes/no), and physical activity (METs min/day), sedentary time (h/day), alcohol consumption in g/day (and adding the quadratic term), food groups (consumption of vegetables, fruits, legumes, cereals, oils and fats, olive oils, biscuits, meat, fish, dairy, nuts [g/day], coffee and tea [mL/day]), at baseline, one, and three years of follow-up were additionally included as fixed effects in the multivariable-adjusted models. Significant values (*p* < 0.05) were highlighted in bold type.

Fig. 2 displays the longitudinal associations (β coefficients and 95% CI) between energy-adjusted dietary vitamin D intake (as continuous) and changes in body composition over the follow-up by intervention groups. Results from linear mixed-effects models with fully adjustment show significant associations between energy-adjusted dietary vitamin D intake and changes in body composition over one year of follow-up in the intervention group but not in the control group. In particular, 1 μg/day higher energy-adjusted dietary vitamin D intake was associated with higher total lean mass (β: 0.10 %; 95% CI: 0.02 to 0.18; P: 0.017) and muscle-to-fat mass ratio (β: 1.00 × 10^−2^; 95% CI: 0.22 × 10^−2^ to 1.78 × 10^−2^; P: 0.011), and with lower total body weight (β: −0.20 kg; 95% CI: −0.34 to −0.05; P: 0.007), total fat mass (β: −0.11 %; 95% CI: −0.19 to −0.02; P: 0.015) and visceral adipose tissue (β: −1.74 × 10^−2^ kg; 95% CI: −3.47 × 10^−2^ to −0.01 × 10^−2^; P: 0.048) at one year of follow-up in the intervention group **(**Fig. 2; Table S3). However, no significant associations between energy-adjusted dietary vitamin D intake and changes in body composition variables were observed after three years of follow-up in the intervention group.

**Fig. 3:**
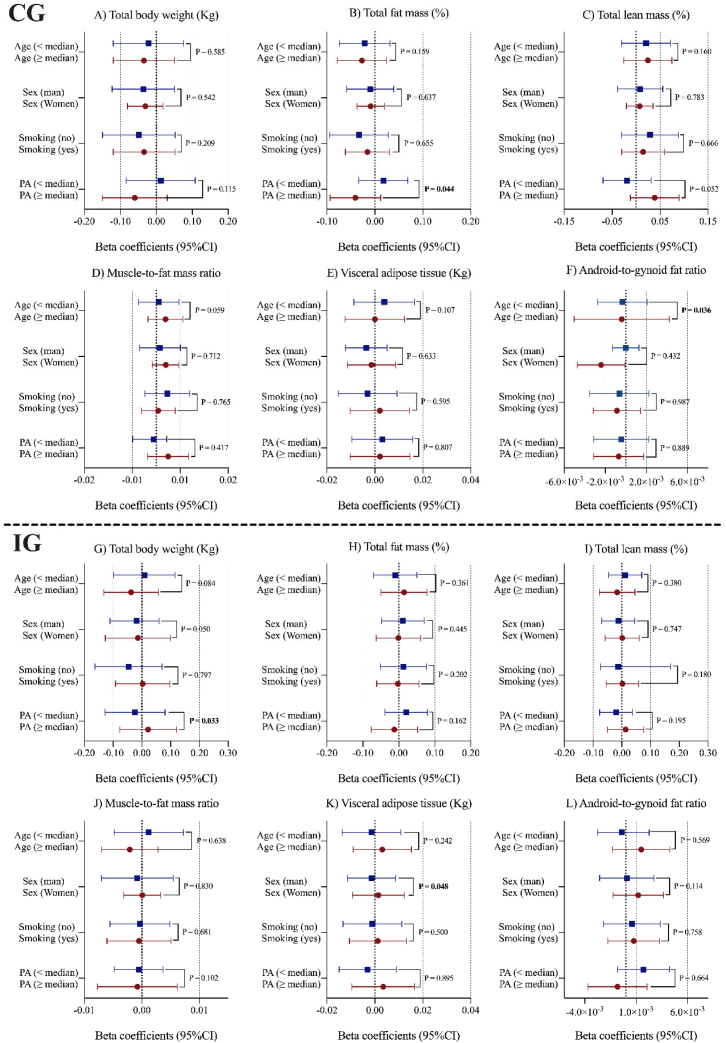
Interaction between energy-adjusted dietary vitamin D intake and different baseline variables of the study potentially related to body composition by intervention groups. Abbreviations: CG, control group; CI, confidence interval; IG, intervention group; PA, physical activity. Three-level linear mixed models were fitted with random intercepts at the recruiting center, cluster family (as couples from the same household were randomized together) and individual participants. An interaction term of energy-adjusted dietary vitamin D intake with time as continuous as well as age (years) and sex (men/women) baseline education level (primary or less, secondary, or college), civil status (single, divorced or separated, married, widower), height (cm), smoking status (current, former, or never), diabetes prevalence (yes/no), hypertension prevalence (yes/no), hypercholesterolemia prevalence (yes/no), and physical activity (METs min/day), sedentary time (h/day), alcohol consumption in g/day (and adding the quadratic term), food groups (consumption of vegetables, fruits, legumes, cereals, oils and fats, olive oils, biscuits, meat, fish, dairy, nuts [g/day], coffee and tea [mL/day]), at baseline, one, and three years of follow-up were additionally included as fixed effects in the multivariable-adjusted models. An interaction term between time, energy-adjusted dietary vitamin D intake, and each potential effect modifier was included within multivariable-adjusted models. Significant values (*p* < 0.05) were highlighted in bold type.

Fig. 3 illustrates the results of the post hoc interaction analyses separately for each study arm between energy-adjusted dietary vitamin D intake and various baseline variables potentially related to body composition. Regarding the control group, significant interactions were found between energy-adjusted dietary vitamin D intake and physical activity for total fat mass (P = 0.044), and with age for android-to-gynoid fat ratio (P = 0.036). In the case of the intervention group, we observed a significant interaction between energy-adjusted dietary vitamin D intake and physical activity for total body weight (P = 0.033), and with sex for visceral adipose tissue (P = 0.048).

## 4 Discussion

The present longitudinal research, which is the first of its kind in the literature, provides significant and novel prospective findings. Total dietary vitamin D intake was observed to be positively associated with better body composition changes in a population with overweight/obesity and metabolic syndrome over a one-year period when following a weight loss and lifestyle intervention which led to modest body composition changes. In particular, positive associations with total lean mass and muscle-to-fat mass ratio, and negative relationships with total body weight, total fat mass, and visceral adipose tissue were observed, even after adjusting for several potential confounders. Collectively, these findings underscore the importance of encouraging vitamin D consumption in older adults through dietary strategies as a feasible approach to potentially ensure optimal body composition changes and its related metabolic benefits in the context of a weight loss and lifestyle intervention leading to body composition changes.

A recent review suggested that dietary or vitamin D medication/supplementation may improve body composition in young adult women in terms of a higher proportion of total lean mass and less total fat mass [11]. However, the importance of vitamin D consumption in the context of weight-loss strategies on body composition has been less explored, and accounts only for supplementation usage [[8], [9], [10]]. In this line, vitamin D consumption via supplements has been reported to be beneficial for weight loss or maintenance, and may lead to a more favorable body composition profile in postmenopausal women under a moderate weight loss situation [8]. Moreover, a sufficient supply of vitamin D has been recommended to reduce the loss of total lean mass under the context of a weight loss intervention for the preservation of total lean mass during induced weight loss among overweight and obese adults [9]. A higher supplementation dosage and a longer intervention period for maintaining sufficient vitamin D status might be required to obtain considerable body weight and composition modifications [10].

In contrast to these studies, we examined the potential significance of dietary vitamin D intake in the context of weight loss among individuals who were not receiving vitamin D supplementation. Under the context of a weight loss and lifestyle intervention, energy-adjusted dietary vitamin D intake was associated with higher increases in total lean mass and muscle-to-fat mass ratio, and higher decreases in total body weight, total fat mass, and visceral adipose tissue. This association was statistically significant at one year (in the active weight loss situation) and remarkably attenuated at three years of follow-up, when weight-loss was only maintained. Our results underscore the importance that dietary vitamin D may have in ameliorating body weight and composition in the context of weight loss and lifestyle interventions. However future randomized clinical trials addressing this research question are warranted.

The current evidence on the significance of dietary vitamin D intake in relation to changes in body weight and composition is both limited and controversial. On the one hand, the association between dietary vitamin D intake and measures of adiposity appears to be more favorable, particularly for the visceral adipose tissue compartment, when subjects fall within a range of low to moderately high adiposity, where variations in visceral adipose tissue are typically more pronounced [16]. Moreover, in the case of total lean mass, a lower dietary vitamin D intake was significantly associated with sarcopenia in older adults aged 50–80 years old [15]. Conversely, on the other hand, dietary vitamin D intake was not significantly associated with sarcopenia incidence or its reversibility in older adults aged ≥65 years. The prevalence of vitamin D deficiency was high, and the dietary vitamin D intake of the participants was not high enough to potentially reverse the situation [13]. In this sense, no relationship between low baseline dietary vitamin D intake and two years of changes in body weight among middle-aged obese individuals was observed, [12] whereas low dietary vitamin D intake was not associated with changes in adiposity in a cohort of adolescents [14]. Dietary vitamin D intake in minimal amounts may not be therefore crucial for relevant changes in body weight and composition [[12], [13], [14]]. In our study, despite the mean dietary vitamin D intake was below reference values (i.e., 10−15 μg/day) [31], the increase in dietary vitamin D intake consumption over time in the intervention group compared to the control group may explain our findings. Furthermore, despite the pervasive issue of insufficient dietary vitamin D intake, attributable in part to the restricted availability of dietary sources rich in vitamin D [32], older adults confronts an augmented additional susceptibility to vitamin D deficiency, which therefore may be encouraged to combine increased intakes of dietary vitamin D with vitamin D supplementation, that may potentially provide additional benefits in terms of body composition changes [33,34].

The proposed mechanisms by which vitamin D consumption could modulate body composition include (a) increased levels of parathyroid hormone that promotes lipogenesis by greater calcium inflow in adipocytes [35], (b) adipogenesis inhibition through vitamin D actions modulated by vitamin D receptors, and (c) reduced adiposity by fatty acid synthase activity suppression [36]. Despite body size and/or adiposity should be taken into account when determining the amount of vitamin D consumption required for optimal status and its potential anti-obesity effects [37], it should be noted that changes in body composition variables are highly multifactorial, and therefore vitamin D is not the only factor underlying body composition modifications [14].

It should be considered that our population was composed by aged overweight/obese individuals. Older adults do not have fully expressed or functional vitamin D hydroxylases, vitamin D receptors, and intestinal absorption of vitamin D [7], and hence, there is a need for increasing dietary vitamin D intake in older adults due to these physiological disturbances [38]. Moreover, obesity per se seems to be associated with lower circulating levels of vitamin D due to low sun exposure, physical activity, and intake of foods rich in vitamin D, as well as a greater volumetric dilution and sequestration in the adipose tissue [39]. Indeed, obese individuals may require 40% higher vitamin D intake than nonobese individuals to attain the same blood vitamin D concentrations [40] to compensate for the lower bioavailability of endogenously produced vitamin D, and hence have the chance to exhibit its potential anti-obesity effects [41]. As increased consumption of vitamin D in subjects with obesity may normalize vitamin D nutritional status accompanied by improvements in body weight and composition [7], and despite dietary vitamin D intake being traditionally considered a minor source, it becomes crucial for older adults and those with excess weight, as they derive significantly less vitamin D from cutaneous synthesis [42,43].

Our study presents notable strengths. First, its longitudinal prospective design facilitated the observation of temporal associations over one and three years of follow-up, although this design does not establish potential causal relationships. Second, body composition was objectively and accurately determined by DXA providing information on total adiposity and its distribution in different tissues and overcame the lack of specificity of anthropometric measures. DXA represents the “gold standard” imaging technique for direct body composition analysis, enabling measures with high-precision, low radiation exposure, and short-scanning time. Lastly, the study benefitted from a large sample size, affording the adjustment of statistical models for various potential confounding factors.

Nevertheless, our study findings should be interpreted in light of certain limitations. Firstly, the potential for reverse causality and residual confounding persists, particularly from unmeasured factors not accounted for in the analyses. Secondly, the generalizability of the results may be limited to older populations with overweight/obesity and metabolic syndrome, precluding extrapolation to other populations. Thirdly, the absence of data pertaining to blood 25(OH)D levels, kidney function, sun exposure, and seasonality represents a notable information gap. Fourthly, measures of body composition were not available in the total cohort, due to limited access to DXA scanners in most of the recruiting centers. Furthermore, reliance on a FFQ to estimate dietary vitamin D intake, despite validated in older Spanish men and women [24], may introduce potential sources of measurement error and recall bias, particularly given its dependency on participants' memory and susceptibility to cognitive decline.

## 5 Conclusion

In conclusion, dietary vitamin D intake is associated with better body composition changes at short term in the context of a weight loss and lifestyle intervention in older individuals with excess weight and metabolic syndrome. Future studies at long term are needed to determine whether higher dietary vitamin D intake above current recommendations should be encouraged in aged individuals following a weight loss and lifestyle intervention approach.

## CRediT authorship contribution statement

Héctor Vázquez-Lorente: Writing – original draft, Writing – review & editing, Methodology, Investigation, Data curation, Conceptualization. Jiaqi Ni: Writing – original draft, Writing – review & editing, Methodology, Formal analysis, Data curation, Conceptualization. Nancy Babio: Writing – review & editing, Methodology, Formal analysis, Data curation, Conceptualization. Ana García-Arellano: Writing – review & editing, Investigation, Data curation, Conceptualization, Funding acquisition. Dora Romaguera: Writing – review & editing, Investigation, Data curation, Conceptualization, Funding acquisition. J. Alfredo Martínez: Writing – review & editing, Investigation, Data curation, Conceptualization, Funding acquisition. Ramon Estruch: Writing – review & editing, Investigation, Data curation, Conceptualization, Funding acquisition. Vicente Martín Sánchez: Writing – review & editing, Investigation, Data curation, Conceptualization. Josep Vidal: Writing – review & editing, Investigation, Data curation, Conceptualization. Montserrat Fitó: Writing – review & editing, Investigation, Data curation, Conceptualization. Maira Bes-Rastrollo: Writing – review & editing. Jadwiga Konieczna: Writing – review & editing. Diego Martinez-Urbistondo: Writing – review & editing. Rosa Casas: Writing – review & editing. Marcos García-Fernández: Writing – review & editing. Romina Paula Olbeyra: Writing – review & editing. Alice Chaplin: Writing – review & editing. M. Angeles Zulet: Writing – review & editing. Jordi Salas-Salvadó: Writing – original draft, Writing – review & editing, Visualization, Validation, Methodology, Investigation, Data curation, Conceptualization, Funding acquisition.

## Ethics approval and consent to participate

The study protocol was approved by the Research Ethics Committees of all recruiting centers. In addition, all participants signed an informed consent form upon entry into the study.

## Funding

This study was supported by the official Spanish Institutions for Funding Scientific Biomedical Research, CIBER Fisiopatología de la Obesidad y Nutrición (CIBEROBN) and Instituto de Salud Carlos III (ISCIII), through the Fondo de Investigación para la Salud (FIS), which is co-funded by the 10.13039/501100008530European Regional Development Fund (six coordinated FIS projects led by JSS and JV, including the following projects: PI13/00673, PI13/00492, PI13/00272, PI13/01123, PI13/00462, PI13/00233, PI13/02184, PI13/00728, PI13/01090, PI13/01056, PI14/01722, PI14/00636, PI14/00618, PI14/00696, PI14/01206, PI14/01919, PI14/00853, PI14/01374, PI14/00972, PI14/00728, PI14/01471, PI16/00473, PI16/00662, PI16/01873, PI16/01094, PI16/00501, PI16/00533, PI16/00381, PI16/00366, PI16/01522, PI16/01120, PI17/00764, PI17/01183, PI17/00855, PI17/01347, PI17/00525, PI17/01827, PI17/00532, PI17/00215, PI17/01441, PI17/00508, PI17/01732, PI17/00926, PI19/00957, PI19/00386, PI19/00309, PI19/01032, PI19/00576, PI19/00017, PI19/01226, PI19/00781, PI19/01560, PI19/01332, PI20/01802, PI20/00138, PI20/01532, PI20/00456, PI20/00339, PI20/00557, PI20/00886, and PI20/01158); the Especial Action Project entitled: Implementación y evaluación de una intervención intensiva sobre la actividad física Cohorte PREDIMED-Plus grant to JSS; the European Research Council (Advanced Research Grant 2014–2019; agreement #340918) granted to MMG; the Recercaixa (number 2013ACUP00194) grant to JSS; grants from the Consejería de Salud de la Junta de Andalucía (PI0458/2013, PS0358/2016, and PI0137/2018); the PROMETEO/21/2021 and the AICO/2021/347 grants from the 10.13039/501100003359Generalitat Valenciana; the Horizon 2020 PRIME study (Prevention and Remediation of Insulin Multimorbidity in Europe; grant agreement #847879); and grant from Agencia Estatal de Investigación (reference CNS2022-135862) to DR. HVL was funded by a research grant from the Agència de Gestió d’Ajuts Universitaris de Recerca via Unión Europea, Next Generation EU (AGAUR, record number: 2023POST-INV-01). JN is supported by a predoctoral grant from Ministerio de Ciencia, Innovación y Universidades (FPU 20/00385). None of the funding sources took part in the design, collection, analysis, interpretation of the data, or writing the report, or in the decision to submit the manuscript for publication. JSS senior author, gratefully acknowledges the financial support by 10.13039/501100003741ICREA under the ICREA Academia program.

## Data availability

Data described in the manuscript, codebook, and analytic code will be made available upon request pending application and approval of the PREDIMED-Plus Steering Committee. There are restrictions on the availability of data for the PREDIMED-Plus trial, due to the signed consent agreements around data sharing, which only allow access to external researchers for studies following the project purposes. Requestors wishing to access the PREDIMED-Plus trial data used in this study can make a request to the PREDIMED-Plus trial Steering Committee chair: jordi.salas@urv.cat. The request will then be passed to members of the PREDIMED-Plus Steering Committee for deliberation.

## Declaration of competing interest

The authors declare that there are no known financial interests or personal relationships that could have appeared to influence the work reported in this paper.
