# Supplementary material for: Dietary vitamin D intake and changes in body composition over three years in older adults with metabolic syndrome
Source: J Nutr Health Aging. 2025 Jan 8;29(3):100467. doi: 10.1016/j.jnha.2024.100467 (PMC12180021; doi:10.1016/j.jnha.2024.100467)
Supplement: Supplementary file 1 [file mmc1.docx]

| **Table S1.-**Baseline characteristics of the PREDIMED-Plus participants (overall and by intervention group). | | | | |
| --- | --- | --- | --- | --- |
|  | **Total population** | **Control Group** | **Intervention Group** | **P-value^1^** |
| Total energy intake (kcal/day) | 2410 ± 529 | 2437 ± 533 | 2381 ± 524 | 0.164 |
| **Nutrients** |  |  |  |  |
| Carbohydrates (En%) | 40.2 ± 6.3 | 40.2 ± 6.5 | 40.2 ± 6.2 | 0.870 |
| Protein (En%) | 16.5 ± 2.8 | 16.4 ± 2.8 | 16.5 ± 2.8 | 0.430 |
| Total fat (En%) | 39.9 ± 6.1 | 39.9 ± 6.1 | 39.9 ± 6.1 | 0.889 |
| Fibre (g/day) | 26.3 ± 8.6 | 26.4 ± 8.3 | 26.2 ± 8.8 | 0.757 |
| **Food groups** |  |  |  |  |
| Vegetables (g/day) | 313.3 ± 122.2 | 314.5 ± 124.0 | 312.0 ± 120.5 | 0.787 |
| Fruits (g/day) | 356.7 ± 207.8 | 357.0 ± 205.8 | 356.3 ± 210.3 | 0.969 |
| Legumes (g/day) | 20.0 ± 10.4 | 19.8 ± 9.4 | 20.2 ± 11.4 | 0.617 |
| Cereals (g/day) | 153.9 ± 79.0 | 157.0 ± 83.2 | 150.7 ± 74.2 | 0.287 |
| Total meat (g/day) | 154.0 ± 59.6 | 152.7 ± 60.7 | 155.3 ± 58.5 | 0.565 |
| Total fish (g/day) | 97.9 ± 46.4 | 98.0 ± 2.4 | 97.8 ± 2.5 | 0.958 |
| Nuts (g/day) | 15.0 ± 0.6 | 15.5 ± 0.9 | 14.4 ± 0.9 | 0.196 |
| Total dairy (g/day) | 331.7 ± 203.6 | 347.2 ± 213.5 | 315.4 ± 191.5 | **0.037** |
| Oils and fats (g/day) | 45.8 ± 17.3 | 46.0 ± 17.8 | 45.7 ± 16.7 | 0.801 |
| Olive oils (g/day) | 42.5 ± 16.2 | 42.4 ± 16.6 | 42.6 ± 15.7 | 0.920 |
| Biscuits (g/day) | 26.0 ± 28.5 | 25.9 ± 29.3 | 26.0 ± 27.6 | 0.964 |
| Coffee and tea (mL/day) | 91.9 ± 63.1 | 92.9 ± 65.9 | 90.9 ± 60.0 | 0.660 |
| Total alcohol (g/day) | 12.5 ± 15.3 | 13.0 ± 14.9 | 12.1 ± 15.7 | 0.461 |
| Data are presented as mean ± SD. Significant values (*p*<0.05) were highlighted in bold type.  ^1^P value for differences intergroups was calculated by unpaired student t-test | | | | |

| **Table S2-** Changes in body composition and energy-adjusted dietary vitamin D intake over one, and three years of follow-up in the PREDIMED-Plus cohort. | | | | | |
| --- | --- | --- | --- | --- | --- |
|  | **Control group (n = 367)** | | **Intervention group (n = 348)** | |  |
|  | Mean ± SD | Mean Δ [min,max] | Mean ± SD | Mean Δ [min,max] | **p-value** |
| **Total body weight (Kg)** |  |  |  |  |  |
| Year 1 vs baseline | 85.3 ± 12.9 | -0.3 [-14.0, 11.7] | 83.9 ± 12.2 | -2.2 [-19.6, 6.4] | 0.001 |
| Year 3 vs baseline | 85.1 ± 13.1 | -0.5 [-17.9, 14.6] | 84.3 ± 12.3 | -1.8 [-20.5, 10.2] | 0.001 |
| **Total fat mass (%)** |  |  |  |  |  |
| Year 1 vs baseline | 38.9 ± 6.5 | -0.2 [-11.9, 5.77] | 38.2 ± 7.2 | -1.2 [-12.5, 5.7] | 0.001 |
| Year 3 vs baseline | 39.1 ± 6.7 | -0.1 [-13.2, 6.9] | 38.9 ± 7.1 | -0.5 [-14.4, 9.9] | 0.015 |
| **Total lean mass (%)** |  |  |  |  |  |
| Year 1 vs baseline | 57.9 ± 6.2 | 0.2 [-5.6, 11.1] | 58.5 ± 6.9 | 1.1 [-5.7, 12.0] | 0.001 |
| Year 3 vs baseline | 57.8 ± 6.3 | 0.1 [-6.7, 12.5] | 57.9 ± 6.7 | 0.5 [-10.0, 14.3] | 0.021 |
| **Muscle-to-fat mass ratio^1^** |  |  |  |  |  |
| Year 1 vs baseline | 1.5 ± 0.4 | 0.1 [-3.3, 13.2] | 1.6 ± 0.5 | 1.0 [-5.4, 19.2] | 0.001 |
| Year 3 vs baseline | 1.5 ± 0.4 | 0.1 [-4.9, 15.5] | 1.6 ± 0.5 | 0.4 [-8.4, 23.7] | 0.025 |
| **Visceral adipose tissue (Kg)^1^** |  |  |  |  |  |
| Year 1 vs baseline | 2.4 ± 0.9 | -0.2 [-26.9, 16.4] | 2.2 ± 0.9 | -1.6 [-31.1, 13.1] | 0.001 |
| Year 3 vs baseline | 2.4 ± 0.9 | 0.1 [-4.9, 15.5] | 2.2 ± 0.9 | 0.4 [-8.4, 23.8] | 0.051 |
| **Android-to-gynoid fat ratio^1^** |  |  |  |  |  |
| Year 1 vs baseline | 0.8 ± 0.2 | -0.0 [-4.4, 3.9] | 0.8 ± 0.2 | -0.2 [-4.7, 5.0] | 0.028 |
| Year 3 vs baseline | 0.8 ± 0.4 | -0.1 [-4.6, 3.9] | 0.8 ± 0.4 | -0.2 [-4.3, 3.9] | 0.253 |
| **Dietary Vitamin D intake** |  |  |  |  |  |
| Year 1 vs baseline | 6.3 ± 3.2 | 0.4 [-9.1, 8.6] | 7.5 ± 3.5 | 1.5 [-12.8, 13.0] | 0.001 |
| Year 3 vs baseline | 6.4 ± 3.5 | 0.5 [-13.5, 12.0] | 7.1 ± 3.6 | 1.1 [-12.8, 13.0] | 0.031 |
| Abbreviations: max, maximum; min, minimum; SD, standard deviation.  Data are presented as mean ± SD, and mean changes (Δ) [min,max] in body composition variables and energy-adjusted dietary vitamin D intake, respectively. Unpaired Student’s t-test was employed to assess differences in mean changes in body composition and energy-adjusted dietary vitamin D intake by study arm.  ^1^Values are expressed as multiples of 10^-1^ (x10^-1^). | | | | | |

| **Table S3.-** Longitudinal associations between energy-adjusted dietary vitamin D intake and changes in body composition over one, and three years of follow-up in the PREDIMED-Plus cohort. | | | | | | | | |  |
| --- | --- | --- | --- | --- | --- | --- | --- | --- | --- |
|  | Basic model | | | | Multivariable-adjusted model | | | |  |
|  | **Control group (n = 367)** | | **Intervention group (n = 348)** | | **Control group (n = 367)** | | **Intervention group (n = 348)** | |  |
|  | **β [95% CI]** | **p-value** | **β [95% CI]** | **p-value** | **β [95% CI]** | **p-value** | **β [95% CI]** | **p-value** |  |
| **Total body weight (Kg)** |  |  |  |  |  |  |  |  |  |
| Year 1 vs baseline | -0.08 [-0.21,0.06] | 0.272 | -0.23 [-0.40,-0.08] | **0.003** | -0.12 [-0.25,0.02] | 0.088 | -0.20 [-0.34,-0.05] | **0.007** |  |
| Year 3 vs baseline | -0.05 [-0.18,0.08] | 0.452 | -0.13 [-0.28,0.02] | 0.092 | -0.08 [-0.21,0.05] | 0.248 | -0.07 [-0.22,0.07] | 0.333 |  |
| **Total fat mass (%)** |  |  |  |  |  |  |  |  |  |
| Year 1 vs baseline | -0.04 [-0.12,0.04] | 0.302 | -0.12 [-0.20,-0.03] | **0.009** | -0.07 [-0.14,0.01] | 0.083 | -0.11 [-0.19,-0.02] | **0.015** |  |
| Year 3 vs baseline | -0.03 [-0.10,0.05] | 0.489 | -0.06 [-0.14,0.03] | 0.208 | -0.05 [-0.12,0.03] | 0.225 | -0.03 [-0.11,0.06] | 0.532 |  |
| **Total lean mass (%)** |  |  |  |  |  |  |  |  |  |
| Year 1 vs baseline | 0.03 [-0.04,0.11] | 0.346 | 0.11 [0.03,0.19] | **0.011** | 0.06 [-0.01,0.13] | 0.101 | 0.10 [0.02,0.18] | **0.017** |  |
| Year 3 vs baseline | 0.02 [-0.05,0.10] | 0.527 | 0.05 [-0.03,0.14] | 0.227 | 0.04 [-0.03,0.11] | 0.254 | 0.03 [-0.06,0.11] | 0.546 |  |
| **Muscle-to-fat mass ratio^1^** |  |  |  |  |  |  |  |  |  |
| Year 1 vs baseline | 0.30 [-0.28,0.88] | 0.312 | 1.07 [0.28,1.85] | **0.008** | 0.47 [-0.09,1.04] | 0.104 | 1.00 [0.22,1.78] | **0.011** |  |
| Year 3 vs baseline | 0.12 [-0.45,0.70] | 0.681 | 0.42 [-0.37,1.21] | 0.297 | 0.24 [-0.32,0.80] | 0.401 | 0.20 [-0.59,0.99] | 0.619 |  |
| **Visceral adipose tissue (Kg)^1^** | |  |  |  |  |  |  |  |  |
| Year 1 vs baseline | -0.99 [-2.82,0.83] | 0.285 | -1.91 [-3.66,-0.15] | **0.033** | -1.45 [-3.26,0.36] | 0.117 | -1.74 [-3.47,-0.01] | **0.048** |  |
| Year 3 vs baseline | 0.38 [-1.43,2.19] | 0.680 | -0.38 [-2.14,1.38] | 0.673 | 0.09 [-1.70,1.88] | 0.925 | -0.37 [-2.12,1.38] | 0.678 |  |
| **Android-to-gynoid fat ratio^1^** | |  |  |  |  |  |  |  |  |
| Year 1 vs baseline | -0.25 [-0.61,0.12] | 0.184 | -0.12 [-0.49,0.25] | 0.529 | -0.36 [-0.72,0.01] | 0.054 | -0.11 [-0.49,0.26] | 0.557 |  |
| Year 3 vs baseline | -0.08 [-0.45,0.28] | 0.651 | 0.08 [-0.29,0.46] | 0.671 | -0.14 [-0.50,0.02] | 0.454 | 0.09 [-0.29,0.46] | 0.656 |  |
| Abbreviations: CI, confidence interval. Three-level linear mixed models were fitted with random intercepts at the recruiting center, cluster family (as couples from the same household were randomized together) and individual participants. An interaction term of energy-adjusted dietary vitamin D intake with time as continuous as well as age (years) and sex (men/women) were included as fixed effects in the basic model. Baseline education level (primary or less, secondary, or college), civil status (single, divorced or separated, married, widower), height (cm), smoking status (current, former, or never), diabetes prevalence (yes/no), hypertension prevalence (yes/no), hypercholesterolemia prevalence (yes/no), and physical activity (METs min/day), sedentary time (h/day), alcohol consumption in g/day (and adding the quadratic term), food groups (consumption of vegetables, fruits, legumes, cereals, oils and fats, olive oils, biscuits, meat, fish, dairy, nuts [g/day], coffee and tea [mL/day]), at baseline, one, and three years of follow-up were additionally included as fixed effects in the multivariable-adjusted models. Significant values (*p*<0.05) were highlighted in bold type.  ^1^β [95% CI] values are expressed as multiples of 10^-2^ (x10^-2^). | | | | | | | | |  |


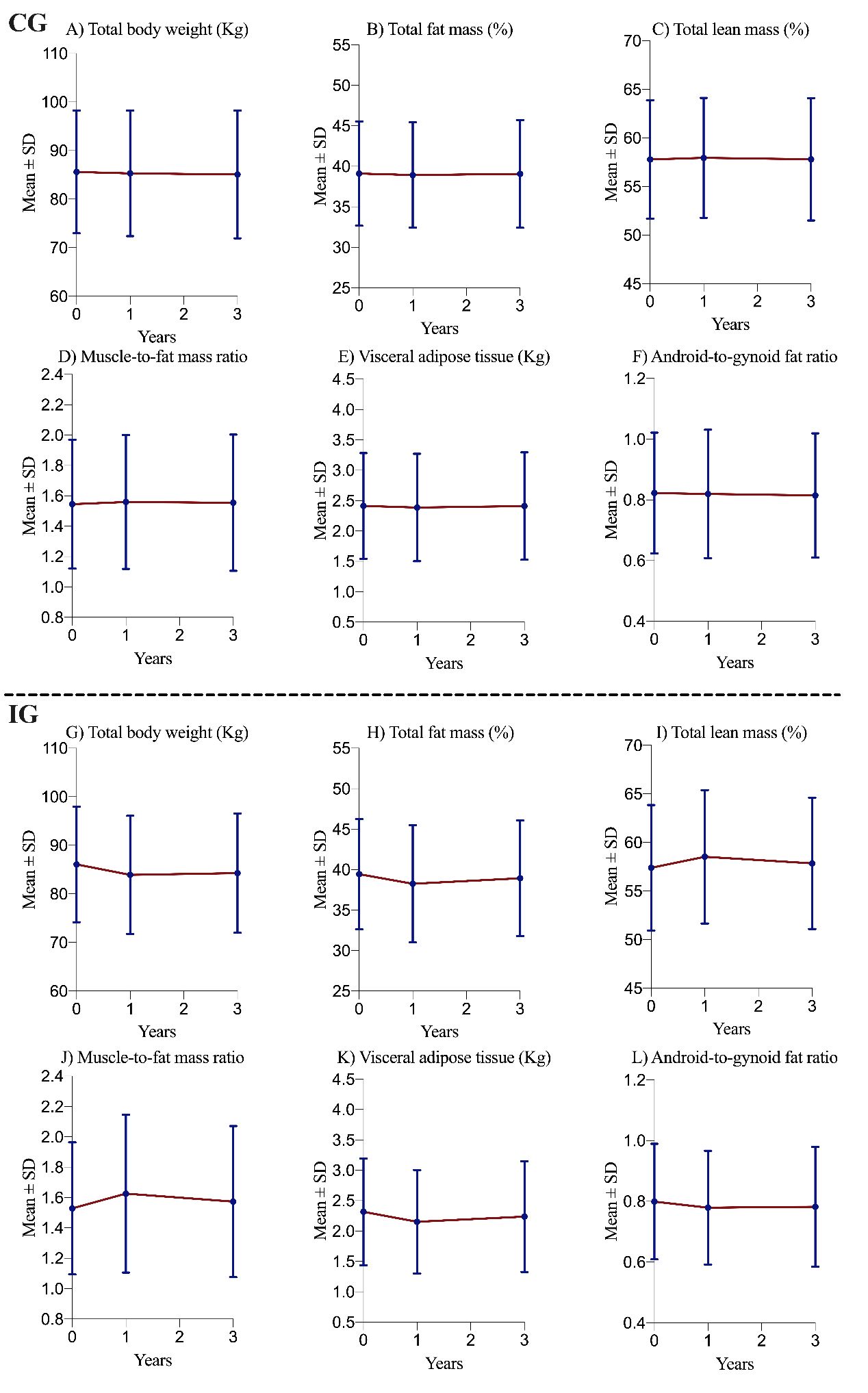


**Figure S1**. **Evolution of body composition variables over one, and three years of follow-up.** Abbreviations: CG, control group; IG, intervention group. SD, standard deviation.





**Figure S2**. **Evolution of energy-adjusted dietary vitamin D intake over one, and three years of follow-up.** Abbreviations: CG, control group; IG, intervention group. SD, standard deviation.
